# AMBIENT: Active Modules for Bipartite Networks - using high-throughput transcriptomic data to dissect metabolic response

**DOI:** 10.1186/1752-0509-7-26

**Published:** 2013-03-25

**Authors:** William A Bryant, Michael JE Sternberg, John W Pinney

**Affiliations:** 1 Centre for Integrative Systems Biology and Bioinformatics, Imperial College London, London, SW7 2AZ, UK

**Keywords:** Metabolic networks, Simulated annealing, High-throughput data, Stress response, Network analysis

## Abstract

**Background:**

With the continued proliferation of high-throughput biological experiments, there is a pressing need for tools to integrate the data produced in ways that produce biologically meaningful conclusions. Many microarray studies have analysed transcriptomic data from a pathway perspective, for instance by testing for KEGG pathway enrichment in sets of upregulated genes. However, the increasing availability of species-specific metabolic models provides the opportunity to analyse these data in a more objective, system-wide manner.

**Results:**

Here we introduce ambient (Active Modules for Bipartite Networks), a simulated annealing approach to the discovery of metabolic subnetworks (modules) that are significantly affected by a given genetic or environmental change. The metabolic modules returned by ambient are connected parts of the bipartite network that change coherently between conditions, providing a more detailed view of metabolic changes than standard approaches based on pathway enrichment.

**Conclusions:**

ambient is an effective and flexible tool for the analysis of high-throughput data in a metabolic context. The same approach can be applied to any system in which reactions (or metabolites) can be assigned a score based on some biological observation, without the limitation of predefined pathways. A Python implementation of ambient is available at http://www.theosysbio.bio.ic.ac.uk/ambient.

## Background

The increasing availability of high-throughput biological data sets promises a deeper understanding of cellular processes than has ever previously been accessible. Many of these high-throughput experiments directly or indirectly measure metabolic changes within a cell, for example microarrays indirectly measure enzyme concentration and metabolomics experiments directly measure changing metabolite concentrations. The metabolism of any organism is a complex system with thousands of interacting entities, meaning that an integrated, network-based analysis is required in order to extract the maximum amount of knowledge from these data.

Common methods for proteome and transcriptome analysis include GO or KEGG enrichment analysis using Fisher’s exact test or gene set enrichment analysis (GSEA [1]). These approaches look for predefined pathways (or gene sets) that are enriched for genes with highly changed transcription (or translation), usually using a two-fold up- or down-regulation cutoff to define these highly changed genes. However, if biologically important pathways cross multiple predefined pathways (or gene sets) they might not be picked up by these approaches, which do not allow flexibility in pathway definitions.

Other approaches have analysed such data using simple protein interaction graphs. Ideker et al. [2] have developed a method called Active Modules, which employs simulated annealing to search for connected components of a protein-protein interaction (PPI) network that are significantly changed according to some microarray dataset, thus giving an insight into how a coordinated response occurs in the PPI network without the need for such predefined pathways or gene-sets.

In order to find significantly changed pathways in metabolism, Breitling et al. [3] developed GiGA (Graph-based iterative Group Analysis), an alternative to the Active Modules approach, which uses a greedy algorithm to build connected components of a gene-gene graph that are significantly enriched with genes having a low p-value for expression change. The chief innovation of GiGA as applied to metabolic network analysis is the ability to link genes by any given set of associations. In [3] these associations are made through metabolites in KEGG pathways, linking genes that encode enzymes sharing a particular metabolite as substrate or product. The gene-gene graph is then a representation of the metabolic network, within which GiGA finds connected components that are enriched for significant gene changes. Such analyses, when applied in the metabolic context, can be thought of as pathway finding methods, uncovering linked modules (or pathways) of genes or proteins that undergo coordinated change as a result of some environmental or genetic perturbation. These modules give a better view of the coordination specifically of metabolic changes and when accompanied by expert interpretation can reveal insights into system-level responses.

Several other recent methods have been developed to take explicit advantage of the underlying metabolic network structure controlled by transcriptional changes in order to determine network-based gene neighbourhoods. PathExpress [4] takes a set of genes of interest (such as genes with p-values for change below a certain cutoff) as an input and for each enzyme finds whether there is enrichment of genes of interest around that enzyme. This ‘enzyme neighbourhood’ is defined through gene-reaction-metabolite relationships in KEGG. However, these neighbourhoods are predefined by the metabolic network before analysis, rather than allowed to take any shape or size. KEGG spider [5] does a similar analysis, but is limited to those organisms covered by the KEGG orthology database. Both of these approaches have similar limitations: they discard information about fold-changes of the entire transcriptome by demanding a significance cutoff for each gene and they also limit the shape and size of inferred modules. Recently Schramm et al. [6] developed a network-topology based approach to finding gene regulatory relationships in the context of metabolism, but this is currently applicable to a limited number of organisms and relies on KEGG annotations of those organisms and KEGG pathway maps.

**Figure 1:**
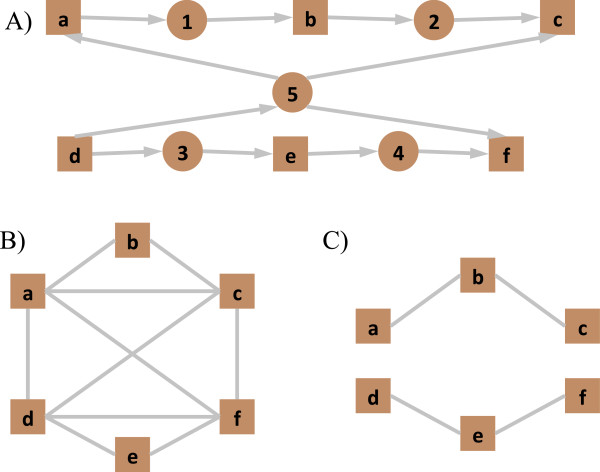
**Differing representations of metabolic networks.** Showing a bipartite representation of two pathways (*abc* and *def*) linked by a highly connected metabolite (I), as used in ambient and a reaction-only representation (II), such as that used in GiGA. In representation **A**, penalising metabolite 5 allows for pathways including only *abc*, or only *def*, as well as pathway crossover if there is sufficient evidence for it. Representation **B** shows how the network would be construed if metabolites were used to link reactions together; the original pathways are lost in this case. Representation **C** shows the network if metabolite 5 were deemed to be a currency metabolite. This retains the two original pathways, but would never link the two pathways even if there were evidence for crossover.

Whilst GiGA and Active Modules can both find arbitrary modules in simple gene-gene interaction graphs, there are several problems in using such graphs to represent metabolic networks. Genes in these networks connect to all other genes associated with a particular metabolite, such that so-called currency metabolites (compounds involved in many reactions, for instance water, carbon dioxide and ATP) can link large swathes of metabolism to create large (complete) subnetworks. Currency metabolites can potentially falsely link unrelated parts of metabolism in pathway-building approaches and must therefore be accounted for. Usually this is done by *a priori* manual removal of these metabolites [4] or simply assigning a maximum connectivity, above which a metabolite will be discarded from the network [3]. However, both approaches require a somewhat arbitrary classification of compounds that may result in the loss of useful information. Figure 1 shows the conceptual problems with these approaches. In the case where a highly connected metabolite is not a currency metabolite, important structural information about the network can be lost by ‘over-connection’ of the network (as in Figure 1B). However, in the case where this metabolite is removed (Figure 1C) the potential for a crossover between the pathways, even supported by experimental evidence, is eliminated.

Genes encoding enzymes with the same function (isozymes) can also be problematic: often the quantity of real interest for a particular reaction is the net change in flux or flux capacity for that reaction, thus multiple gene changes related to a single reaction should be accounted for together rather than as separate nodes in a graph representation of metabolism. In contrast, multi-function enzymes can potentially erroneously link two separate parts of metabolism and cause inferred modules to consist of otherwise disconnected pathways within the metabolic network.

The bipartite metabolite-reaction representation of metabolism solves all of these problems by representing both metabolites and reactions as nodes and mapping data onto these entities separately. Figure 1A shows such a bipartite network (circles representing metabolites and squares representing reactions). This representation allows the full relationship between reactions and metabolites to be used in the investigation of metabolic changes. No metabolites need be removed from the initial network because they can be individually assessed for inclusion in any pathway prediction (in conjunction with the ambient scoring system as detailed in the Methods section). Since reactions, rather than genes, are used for mapping data onto the network, isozymes and multifunctional enzymes can be amalgamated and separated respectively according to actual enzymatic function (also in conjunction with the ambient scoring system), eliminating the problems of gene-centric metabolic network representations.

The analysis of such a network representation requires that there is information mapping genes to reactions. Until recently this would have limited the use of this approach to organisms with published manually curated metabolic models (for example [7]) and organisms that are present in such metabolic network databases as KEGG [8] and BioCyc [9], many of which are not subject to any curation. However, Henry et al. [10] have implemented a system to automatically reconstruct a draft-quality metabolic model for any prokaryotic organism with a complete genome sequence, thus enabling bipartite metabolic networks with gene-reaction mappings to be produced for any of these organisms.

In this paper we introduce ambient, a new method for high-throughput data analysis in the context of metabolic models. ambient is an extension of the Active Modules approach such that it accommodates bipartite (reaction and metabolite) networks, allowing coordinated metabolic pathway changes to be discovered from transcriptomic or metabolomic data. Simulated annealing is used to find modules (i.e. connected components) containing reactions associated with genes that are highly changed in expression and metabolites that have low overall connectivity (low weights, after Croes et al. [11]).

ambient has several advantages over previous approaches to the discovery of metabolic modules. The bipartite representation of metabolism offers a systematic objective representation of metabolism which enables unbiased pathway finding. It also treats all metabolites equivalently, so information about the metabolic network is not lost through arbitrary decisions about which metabolites should be considered currency metabolites. In this paper we compare ambient to GiGA [3] to show how it complements and improves on existing approaches to metabolic module finding in high-throughput data analyses.

## Methods

The algorithm implemented in ambient is based on the Active Modules approach of Ideker et al. [2], extended to a bipartite network. The largest modification of the approach apart from the changed network representation is to toggle edge membership rather than node membership in candidate subnetworks, as described below. A Python implementation of ambient is provided at http://www.theosysbio.bio.ic.ac.uk/ambient.

### Input data and outputs

ambient requires as input a metabolic model and scores for those reactions for which data is available. By default, metabolite scores are calculated using their degree in the metabolic network (as described below) so their scores need not be specified by the user. For models with gene-protein-reaction (GPR) relationships, scores for genes can be provided, which are converted into scores for reactions. The metabolic model is represented by an undirected bipartite network, where an edge links a reaction node with a metabolite node when the metabolite is either a substrate or product of that reaction. Scores for reactions for which there are no data are by default set as the median score of the set of known reaction scores.

The Python implementation of ambient outputs three files: a ‘.tsv’ flatfile results table of all nodes from each significant module, a GraphML file for results visualisation and a ‘.dat’ file in the Python shelve format containing all input and output variables.

### Scoring nodes

Reaction nodes must each be assigned a score, according to the data being analysed. Although in principle this can be based on any dataset, in this work we have investigated the use of ambient with microarray data, so reactions have been scored according to the transcriptomic data for the enzymatic genes known to be involved in the catalysis of those reactions. The scores of each reaction have been assigned in the following way: where a reaction is linked to a single gene in its GPR the reaction is given the value of the fold-change of that gene’s transcript.

Where a reaction is catalysed by a single enzyme made up of multiple different gene products the mean of the fold-changes of those genes is assigned to the reaction. In this way error propagation from individual gene transcript measurements is reduced. Without any knowledge of relative activities of enzymes for each reaction, if a reaction is catalysed by multiple enzymes the mean of the inferred enzyme fold-changes is used. This takes into account all of the enzyme level changes affecting single reactions in a single fold-change score.

### Scoring modules

A module *m* is defined as the union of a subset *r*^*m*^ of reactions and a subset *c*^*m*^ of metabolites that subtend a connected subgraph within the bipartite metabolic graph. The score *S* of a module is given by

$$S(m)=\ln(q)\left(\underset{i}{\sum }s(r_{i}^{m})-\alpha \underset{j}{\sum }w(c_{j}^{m})\right),$$

where *q* = |*r*^*m*^| + |*c*^*m*^| is the number of nodes in the module *m*, $s(r_{i}^{m})$ is the score of the *i*^th^ reaction in the module, $w(c_{j}^{m})$ is the weight (the degree in the original network) of the *j*^th^ metabolite in the module and *α* is a constant given by

$$\alpha =\frac{\left|c|\underset{i}{\sum }s(r_{i}^{+}\right)}{\left|r^{+}|\underset{j}{\sum }w(c_{j}\right)},$$

$$\text{where}\;\;r^{+}\subseteq r:s(r_{i}^{+})>0\;\forall \;r_{i}^{+}\in r^{+}.$$

*α* is defined here as the mean score of all reactions for which *s* > 0, divided by the mean value of *w* over the complete network. This formula for *α* is chosen to ensure that modules are not needlessly restricted by metabolite weights, but do not extend to cover large, biologically meaningless regions of the network. This algorithm tracks all module scores, the logarithmic term being added to bias exploration towards larger modules, without generating extremely large, biologically meaningless modules.

As in the original Active Modules algorithm, the aim is to find the set of non-overlapping modules with the highest total score. Finding global maximum scoring modules is NP-Hard [2], so a simulated annealing approach is taken. In order to search the space of all modules efficiently, a random set of edges (rather than a single edge) is toggled at each step of the simulated annealing.

In many cases, modules describing downregulated regions of the network are of as much interest as upregulated regions. These modules can be investigated by multiplying reaction scores by -1, which gives the most reduced reaction the highest score, and leaving the metabolite scores negative. With this transformation, downregulated modules can be found using the same algorithm as upregulated ones.

### **Ambient** algorithm

The algorithm for finding high scoring modules is outlined here (for a complete description of the method, see the Additional file 1): 

1. Set *t* = *t*_*init*_, *t* = *t*_*init*_,

2. Select a random set of edges, *E*, of size *t*, as the initial edge set,

3. Calculate the score $\sigma (E)=\underset{k}{\sum }S(m_{k}^{E})$, where *m*^*E*^ is the set of connected components induced by *E*,

4. Select *t* edges at random to form the toggle set, *F*,

5. Propose a new edge set, *P* = *E* ∪ *F* - *E* ∩ *F*,

6. Calculate $\sigma (P)=\underset{k}{\sum }S(m_{k}^{P})$,

7. If $\rho <e^{\frac{\sigma (P)-\sigma (E)}{T}}$, where *ρ* is a random number drawn from [0,1), accept the proposed move and set E = P,

8. Repeat steps 4 to 7 until the temperature reduction criterion (see below and Additional file 1) is met,

9. Set *t* = 0.9*t* and *T* = 0.9*T*, 

10. If the maximum number of annealing steps is reached then END, otherwise go to step 3.

Here, *t* is the number of edges to toggle during any one toggle step and *T* is the effective temperature for annealing, determining the tolerance for accepting negatively scoring steps. *t*_*init*_ and *t*_*init*_ are determined from the number of nodes and node scores respectively, as described in the Additional file 1. The temperature reduction criterion is based on adaptive simulated annealing, giving a highly efficient cooling schedule. For any particular temperature, annealing steps are taken until the system reaches thermal equilibrium (in this case when the sum of the scores of the modules over, say, 5000 steps does not change by more than 5%) and this represents the temperature reduction criterion. If this is met, or a cutoff number of steps is reached (i.e. the score fails to converge over a long period) then the algorithm moves to step 9.

### Determining module statistical significance

The significance values of modules found by ambient are found empirically because the distribution of module scores for different sized modules is not known *a priori*. For each module, the number of reactions and metabolites contained in that module are determined. A random sample of scores for that number of reactions and metabolites is summed to produce a random score for a module of that size. This is repeated *P* times (set by the -P option at the command line, default =10000) and the empirical p-value is equal to the number of scores in this sample greater than the module score from annealing divided by *P*. This is determined for the top *M* modules. These *M* p-values are corrected for multiple testing using the Benjamini-Hochberg correction.

### Module visualization

All network diagrams were created using Cytoscape v2.8.2 [12].

## Results and discussion

### Case study: diauxic shift in *Saccharomyces cerevisiae*

The data used to validate the ambient tool were taken from an experiment by DeRisi et al. [13], investigating the transcriptional response of *S. cerevisiae* to glucose depletion. The gene scores used were the log fold-changes between the 0 hour timepoint (glucose rich) and 20.5 hour timepoint (glucose starved) of that experiment. The YEASTNET yeast model [14] was taken as the basis metabolic network. This network is available in SBML format so could be used directly by ambient, and was also converted into a simple protein interaction network with edges induced by shared metabolites for input to GiGA [3].

Overall the DeRisi dataset covers 6153 genes, of which 903 are present in the YEASTNET model, which through the gene-reaction relationships in that model provide scores for 1239 of the 1865 reactions in the model. Since the overall change in metabolism was being investigated, those reactions for which there were no data were assigned the median score of the reactions with known scores. Due to the formulation of the method, ambient modules may include reactions for which there is no data, as long as the scores from the surrounding reactions support such an inclusion.

ambient was run with all parameters set as default except the maximum number of annealing steps *N*, which was set to 1,000,000. GiGA was run with all parameters at default values, ignoring metabolites with degree greater than 20 (currency metabolites) and restricting the number of genes per module to 40. Since GiGA does not make predictions about which metabolites are in particular modules, a simple rule was followed: where any non-currency metabolite was connected to more than one reaction in a GiGA module, it was included in that module. The analysis was run twice for each method, to find both up- and down-regulated modules; of particular interest was whether the main result of [13], of the rerouting of carbon in the Glycolysis/Gluconeogenesis (GLY/GNG) pathway and the TCA cycle, were recapitulated with this metabolic model.

**Figure 2:**
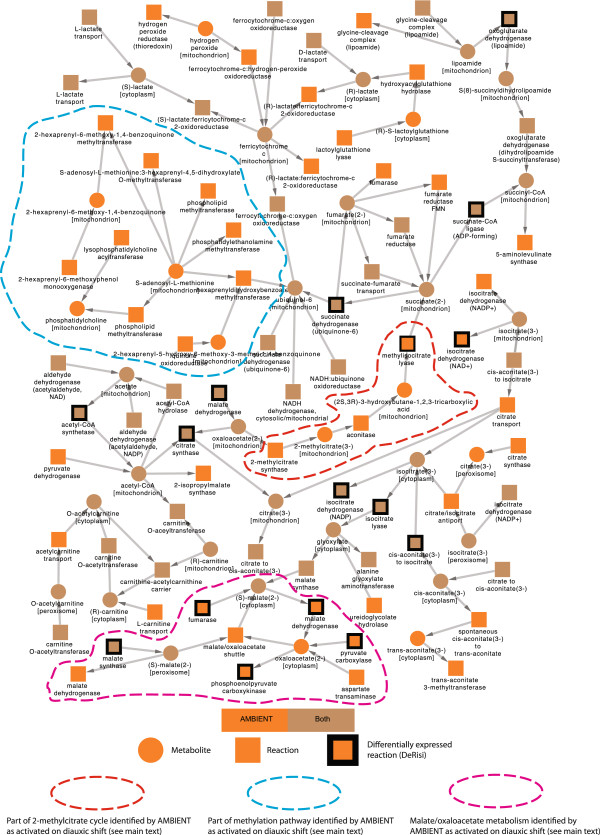
**Up-regulated module 1 from ****ambient**** analysis over yeast diauxic shift.** Showing the complete set of reactions and metabolites inferred by ambient to be present in the highest scoring (and largest) coordinately changed part of metabolism. Reactions outlined in black are indicated in the DeRisi analysis of the same data to be the upregulated part of metabolism responsible for carbon rerouting in central carbon metabolism due to the diauxic shift induced in that experiment [13]. Orange coloured nodes are only found in the ambient module, whilst those coloured brown are also present in a module returned by GiGA. Metabolites in different compartments are explicitly treated as different metabolites in metabolic models such as this, so can be seen more than once (along with an indication of the compartment for each one).

Table 1 summarises the results obtained from GiGA and ambient. The ambient analysis in both directions produced many positive scoring modules, of which four were significantly up-regulated and one was significantly down-regulated at the *q* < 0.05 level (all of these had q-values of < 10^-4^). The highest weight (connectivity) metabolite found to be in one of these modules is cytoplasmic L-glutamine, connected in the full metabolic network to 19 reactions. Figure 2 shows the top up-regulated module and Additional file 2: Figures S1 (up-) and S2 (down-) show all of the other modules found by ambient, along with their overlap with the GiGA modules found. The corresponding diagrams for GiGA are in Additional file 2: Figures S3 and S4. Additional file 2: Figure S2 shows the very large single ambient down-regulated module consisting of 333 reactions and metabolites. This large module indicates that the general downshift of metabolism between hour 0 and hour 20.5 in the experiment is captured in the ambient analysis. The module shows the reduction of lipid biosynthesis, amino acid production and also includes the biomass production reaction itself, as well as picking out the GLY/GNG pathway discussed below.

**Table 1:** **Summary comparison between**ambient**and GiGA**

	**ambient**	**GiGA**
No. of significant modules	4	6
No. of reactions in significant modules	164	220
Q-value of top module	<10^-4^	<10^-50^
No. of reactions in top module	112	75
**Comparison**		
F-measure (significant modules)	0.363	
F-measure (top modules)	0.461	
Adjusted Rand	0.288	

Summary statistics comparing the GiGA and ambient approaches to analysing the diauxic shift of *Saccharomyces cerevisiae* on glucose starvation.

The yeast model used is compartmentalised, so mitochondrial and cytosolic reactions and metabolites are separated. Both GiGA and ambient recapitulate the main conclusions of previous analyses [3,13], namely that the most significantly affected parts of metabolism are the TCA cycle and associated respiratory chain complexes. They also both capture the TCA cycle in the mitochondrial compartment, as expected in yeast.

Each of the three parts of the central carbon metabolism show up in different modules in the ambient analysis. Detailed results for these pathways can be found in Table 2 which shows inferences for ambient, GiGA and from expert manual inspection [13]. This table shows the extent to which each computational method recapitulates the manually observed carbon rerouting through central metabolism in the diauxic shift induced in *S. cerevisiae*. To assess the similarity between the two computational approaches and the DeRisi analysis, the recall, precision and F-measure were calculated, with the results as shown in Table 3. These data indicate that ambient agrees somewhat better with expert interpretation of data for both the GLY/GNG top pathway and the TCA cycle results. Whilst recall is much better for the TCA connection pathway, ambient has a very poor precision. This reduced precision is because the down-regulated pathway found by ambient is very large, reflecting the general downshift in metabolism rather than just the most highly repressed enzymes. As seen in Additional file 2: Figures S3 and S4, each of the GiGA modules is separated into unconnected modules from the conversion between the simple gene-gene evidence graph used in the analysis to the bipartite metabolite-reaction representation. This is due to genes being involved in more than one reaction, where metabolites do not necessarily overlap between these reactions.

**Table 2:** Details of the reactions regulated in diauxic shift in the GLY/GNG pathway and the TCA Cycle

**Loci**	**ID**	**Value**	**ambient**	**GiGA**	**Diff. expressed**
			**Module**	**Module**	**Reactions(DeRisi)**
**Glycolysis/Gluconeogenesis - top**					
NTH1,2, ATH1	2868	1.87	u2	u2	1
TPS1,2, TSL1, TPS3	2870	1.21	0	u2	1
GSY1,2, GLG1,2	3204	2.35	u2	u2	1
GLC3	2663	2.87	u2	u2	1
UGP1	3729	1.01	u2	0	1
GDB1	3154	2.6	u2	u2	1
PGM1,2	3518	1.56	u2	u2	1
PGI1	3160	0.48	0	0	0
GPH1	3205	0.43	u2	0	0
FBP1	3140	3.84	0	u2	1
HXK1, GLK1, HXK2	3230	0.18	0	u2	1
**TCA Cycle**					
PCK1	3514	3.88	u1	0	1
PYC1,2	3594	1.59	u1	0	1
PDA1,2, PDB1, LPD1, PDX1	3597	0.1	u1	0	0
ACS1,2	2785	3.7	u1	u1	1
ALD2	2860	2.25	0	0	1
CIT1	2985	2.72	u1	u1	1
ACO1	2965	2.63	u1	u1	1
IDH1,2	3286	1.49	u1	0	1
KGD1,2, LPD1	3462	2.17	u1	u1	1
LSC2	3660	0.71	u1	u1	1
SDH1,2,3,4	3658	2.53	u1	u1	1
FUM1	3142	1.89	u1	0	1
MDH1	3346	2.57	u1	u1	1
IDP2	3287	3.27	u1	u1	1
ICL1	3290	3.7	u1	u1	1
MLS1	3349	3.22	u1	u1	1
MDH2	3345	1.38	u1	0	1
**GLY/GNG - TCA connection pathway**					
PFK1, PFK2	3516	-1.02	d1	0	-1
FBA1	3010	-1.25	d1	0	-1
TPI1	3698	-1.12	d1	0	-1
TDH1,2,3	3182	-0.55	0	0	0
PGK1	3522	-0.51	0	0	0
GPM1	3523	-1.72	d1	0	-1
ENO1,2	3055	-1.18	d1	0	-1
PDC1,5,6	3595	-1.91	0	d1	-1
PYK1	3598	-1.81	d1	d1	-1

A list of genes in central carbon metabolism that are involved in the regulated response to diauxic shift (identified in [13]) and the reactions for which they encode enzymes, along with their inferred transcriptional changes (Value, mean log fold-change). ID represents ID of the relevant reaction node in the ambient network. The final three columns represent which genes and reactions are up- or down-regulated according to ambient, GiGA and [13] (expert curation). The ambient and GiGA columns indicate which modules the reactions are in and the the Identified Reactions column shows those curated manually. ‘Diff.’ stands for ‘differentially’.

**Table 3:** A statistical comparison of central carbon metabolism modules

	**ambient**	**GiGA**
**Glycolysis/Gluconeogenesis - top**		
Module	u2	u2
Recall	.67	.89
Precision	.33	.23
F-measure	.44	.36
**TCA Cycle**		
Module	u1	u1
Recall	.94	.63
Precision	.19	.13
F-measure	.32	.22
**GLY/GLN - TCA connection pathway**		
Module	d1	d1
Recall	.86	.29
Precision	.03	.05
F-measure	.05	.09

Values of recall, precision and F-measure for the comparison between the two computational inference methods for metabolic modules and the expert curation of DeRisi et al. [13]. The modules containing the relevant parts of metabolism for each computational method are indicated on the table, where ‘u’ and ‘d’ represent up- and down-regulated modules and the number corresponds to the numbers of the modules found in order of significance by the two methods. Diagrams for these can be found in the Additional file 2 except ambient u1, which can be seen in Figure 2.

### Additional insights from **ambient**

In addition to finding the important parts of metabolism discovered by previous methods, ambient finds several linked but previously undiscovered pathways which add to our appreciation of the full metabolic response of *S. cerevisiae* to glucose depletion and diauxic shift. It is clear from the upregulated modules (Figure 2 - module u1, Additional file 2: Figure S1 - modules u2 to u4) that ambient finds the important changes in the TCA cycle and respiratory chain complexes discovered previously by GiGA. Additional file 2: Figures S1-4 show the parts of metabolism identified by ambient that connect to and between the GiGA modules found and the overlap between ambient and GiGA pathways. There are several pathways picked out by ambient that show some expression change and link between the metabolic pathways that have already been identified by others [3,13] to change on diauxic shift in yeast. This indicates a larger scale coherent metabolic response than has previously been recognised.

ambient identifies a further pathway linking mitochondrial oxaloacetate to succinate (central orange pathway in Figure 2, outlined in red) which forms part of the 2-methylcitrate cycle, converting propionate to pyruvate. Additional file 2: Figure S1A shows that in addition to the glycogen metabolic pathways, galactose metabolic pathways are also activated. S-Adenosyl-L-methionine is important for DNA methylation and although *S. cerevisiae* has little methylation in its regulation, Figure 2 shows that metabolism around this chemical is activated on diauxic shift (outlined in blue). Malate and oxaloacetate metabolism also show a coordinated increase (outlined in pink). Overall, these results show that there is a considerable response to glucose starvation in yeast, and ambient has found modules that extend beyond the boundaries of conventionally accepted pathways.

### Differences between **ambient** and GiGA

It can be seen that there is a large overlap between the results of the two algorithms compared here, however the ambient algorithm has found some additional changed reactions that appear to be part of the coordinated response of *S. cerevisiae* to glucose starvation. GiGA identifies some reactions that are not in significant ambient modules. In the upregulated results it finds many transport reactions, though these are mostly disconnected from the rest of the GiGA modules, so do not appear to be parts of coordinated pathways at the transcriptomic level. The cytosolic pentose phosphate pathway is identified by GiGA (Additional file 2: Figure S3B) as being up-regulated, which indicates a potential limitation of ambient’s pathfinding ability, though being a small part of a larger metabolically disconnected module the significance of the transcriptional change of that part by itself is not known. This is also true of the top down-regulated module identified by GiGA (Additional file 2: Figure S4A) which contains several small pathways absent from ambient, but for which there are not individual significance values.

The use of metabolite information is important in metabolic network analysis. Highly connected metabolites have limited utility for constructing modules of coordinated responses to metabolic and environmental perturbations because they carry little information about the flow of material through the metabolic network. It can therefore be asserted that for the purposes of pathfinding, links in metabolic modules through more highly connected metabolites are less significant than those through metabolites connected to fewer reactions. GiGA deals with this problem by excluding a predefined set of currency metabolites, whilst in ambient the relative weights in the metabolic network are taken explicitly into account. In the analysis presented here, the two approaches appear to be somewhat equivalent, as the highest degree metabolite node in the ambient results has a degree of 19.

The advantage of mapping gene scores onto reactions and finding reaction-metabolite modules rather than protein-protein modules is apparent when looking at the relationship between reactions and genes. Approximately half of all genes in the yeast model are associated with more than one reaction, and some are associated with many reactions. For instance, gene YKR009C (FOX2) which is present in GiGA module 3 encodes a multifunctional enzyme associated with 29 reactions in the yeast model. Using GiGA (or any analysis method relying on a gene- or protein-centric representation of metabolism) will fail to distinguish between potentially very different reactions catalysed by such multifunctional proteins and will infer either all or none of them as parts of a metabolic module. This approach will therefore always lack the specificity inherent in the reaction-metabolite bipartite network representation of metabolism and results in unconnected ‘modules’, as seen in Additional file 2: Figures S3-4. A number of the reactions associated with YKR009C are in modules returned by the ambient analysis, but none of these modules are statistically significant.

Another important advance in ambient over previous approaches to module-finding is that it encourages larger modules to form which might otherwise be hidden due to individual interconnecting low-scoring (or negative scoring) nodes or due to missing experimental data. This advantage, inherited from the simulated annealing approach, means that a fuller picture of how metabolic responses are coordinated can appear from the same data used in previous approaches, as can be seen in the good coverage of previously known and newly discovered affected parts of metabolism shown in Figure 2.

Against these advantages, it should be noted that the GiGA approach is much faster than ambient in this case study, taking approximately 30 seconds on a 2.4 GHz chip, as opposed to ambient’s two hours.

### Consistency between **ambient** runs

Since ambient is not a deterministic algorithm, it arrives at slightly different solutions each time it is run. ambient was run ten times with the settings and data described above and the the consistency of the results was assessed. Each run produces what is in effect a partition of the set of all nodes, with each partition being a significant module, except the largest partition which consists of all nodes not in significant modules. The Adjusted Rand index [15] was used to quantify the similarity of results. The ten runs compared with each other produced an Adjusted Rand value of 0.71±0.07, indicating highly consistent results between runs, despite the stochastic nature of the algorithm.

### Limits to the discovery potential of **ambient**

While ambient is very effective for elucidating metabolic changes from transcriptomic datasets it does have several limitations that should be borne in mind. Firstly it does not in any way take into account regulation except in as much as the regulation is apparent in the changes of transcription levels of genes encoding enzymes. Secondly, as with all other current methods for mapping transcription onto metabolic networks, if genes with enzymatic products are not mapped onto the metabolic network then information about their changes cannot be used to investigate the changes in metabolism. Due to this inability, ambient does not extract all of the information possible from transcriptomic data, but just the information from genes with known functions. The elucidation of metabolic functions for uncharacterised genes would therefore require a different approach.

The best way to interpret transcriptomic changes in terms of their effect on reaction rates is an open question. With the approach to reaction scoring presented here, mismapping between mRNAs (transcripts) and reactions could impact on the effectiveness of ambient. Also the best way in which transcript values are combined for each reaction is not known. If some knowledge of the relative activity of various enzymes for each reaction were integrated into this approach a more refined way of combining enzyme inferred changes could be used to score each reaction. Despite these potential limitations ambient has recapitulated the results produced by a very different computational approach and by expert curation of the same data used here, so some confidence can be drawn concerning the scoring system presented here.

### Potential applications and future developments

ambient has been applied here to transcriptional data looking at yeast diauxic shift. This approach is of course applicable to any dataset containing two (or more) microarray measurements in differing conditions. However, due to the information now available in many metabolic models, and the use of a bipartite representation of metabolism, any information that can be mapped to genes, enzymes, reactions or metabolites can be included in an ambient analysis. The explicit links available between genes, proteins and reactions means that transcriptomic (as shown here), proteomic or fluxomic data could be mapped onto the metabolic network under study. Furthermore, metabolites were scored here purely by weight in the metabolic network, but if metabolomic information were available this could easily be incorporated into the scoring system.

In order to look at the overall changes in yeast diauxic shift, the scores for reactions were left as mean log fold-changes of gene transcription. In order to further dissect any transcriptomic dataset, it would be possible to set the median reaction score to zero, therefore just search for especially up- and down-regulated pathways.

Future developments will include methods for combining several different datasets into a single module-finding problem. Since the metabolic models used for this analysis can also be used in flux balance analysis (FBA), methods to combine computational flux estimates obtained from FBA with experimental data will be of particular interest in order to extend and refine our understanding of metabolic network function.

## Conclusions

In this paper we have presented ambient, a method for finding metabolic modules that represent coordinated metabolic differences between two environments or conditions. Using simulated annealing, ambient can go beyond previous approaches to the system level analysis of metabolic changes, as demonstrated here using transcriptomic data from a yeast glucose runout experiment. The bipartite representation of metabolism allows a much greater specificity of analysis and can incorporate information about metabolites, giving an objective view of the changes in cells during the diauxic shift.

This approach has the potential to integrate many different types of high-throughput data obtained in a single experiment. It is hoped that ambient will enable the analysis of disparate data that would otherwise be difficult to integrate, thus improving the biological understanding accessible using high-throughput experimental techniques.

## Competing interests

The authors declare that they have no competing interests.

## Authors’ contributions

JP and MS conceived the project, WB and JP developed the tool and WB wrote the manuscript. All authors read and approved the final manuscript.

## Supplementary Material

Additional file 1 **Supplementary Methods.** Further details on the ambient algorithm and scoring system. Click here for file

Additional file 2 **Supplementary Figures.** Figures S1-S4: diagrams of the positive and negative modules found by both ambient and GiGA. Click here for file
