# Supplementary material for: AMBIENT: Active Modules for Bipartite Networks - using high-throughput transcriptomic data to dissect metabolic response
Source: BMC Syst Biol. 2013 Mar 25;7:26. doi: 10.1186/1752-0509-7-26 (PMC3656802; doi:10.1186/1752-0509-7-26)
Supplement: Additional file 1 — Supplementary Methods. Further details on the ambient algorithm and scoring system. [file 1752-0509-7-26-S1.pdf]

AMBIENT: Active Modules for Bipartite Networks. Using high-throughput transcriptomic data to dissect metabolic response.

Supplementary Methods

Input data and outputs

`ambient` requires two input files to run - a metabolic model stored in SBML format [1, 2], and a tab-separated file containing scores for each reaction, with reactions labelled according to their ID in the SBML model. In principle, reaction scores can come from any source, but in our case study transcriptional data are used. Any metabolic model stored in the SBML format can be used by `ambient` to investigate metabolic changes as mediated by transcription change, as long as gene associations for reactions are given in the model.

A bipartite network is extracted from the SBML file using libSBML [3] and a NetworkX [4] digraph object is created to hold this network. Reactions and metabolites are all represented as nodes. An edge connects a metabolite to a reactions when that metabolite is either a substrate or a product of that reaction. Information about the directionality of the reactions is retained only to indicate which metabolites are co-produced or co-consumed by particular reactions, this information is not used in the analysis itself. Information is also extracted about GPR (gene-protein-reaction) relationships, which can be used for calculation of reaction scores based on the scores of their encoding genes.

Reaction scores from a separate file are then mapped to the relevant reactions in the metabolic network. In the examples shown, mean log fold-change of the genes associated with each reaction was used as the score, and for reactions without a score (those not linked with any genes that have transcriptional data associated with them), the score was set to the median score.

When `ambient` is run from the command line it produces three files: a .dat file created by the Python built-in shelve module, containing all of the relevant output Python objects, a GraphML file containing the metabolic network in bipartite form and an attribute for each node indicating its module membership as determined by `ambient` and a flatfile table listing the reactions present in each of the significant modules found at the end of the simulated annealing run.

`ambient` algorithm

See methods in the main text for a description of the simulated annealing algorithm.

Initially, $1/50$ (t_{init}) of the available edges are proposed at random and modules induced by those selected edges are found and scored. Initial results and timings indicated that several improvements could be made to the simulated annealing strategy of Ideker et al. [5]. This paper suggested retaining modules with the top 20 scores for improved annealing, but because the program must find and score all modules anyway, it is computationally inexpensive to keep track of many more modules at each step. The default maximum number tracked in this program is 1000, though in practice in metabolic networks it would be very unlikely to reach this number of modules.

In order to search the space of all modules efficiently 'toggling' of sets of edges (rather than individual edges) are done by selecting a set number of edges at random and either removing them from or adding them to the proposed set, as appropriate. After each step, all modules induced by the new proposed edge set are scored and ordered, then compared with the scores from the previous proposed edge set. The decision on whether to keep the proposed edge set is done according to [5], by comparing the two ordered sets of module scores to determine increases or decreases in those scores.

Importantly, if an edge toggle causes a drop in any of the scores, it is not necessarily discarded. There is a chance the toggle is kept anyway, the probability being inversely proportional to the score difference multiplied by the temperature T. For computational efficiency, an adaptive cooling schedule has been adopted. The principle behind the schedule is that analogously to real annealing, the network should reach thermal equilibrium at each temperature as it cools in order to find the best scoring modules in the least computational time. In effect, this means that at each temperature steps are taken until the increase or decrease in score over the previous 1000 steps is below a certain threshold, then the temperature is dropped by a constant factor of 0.9 and simultaneously the number of edges toggled per step reduces by the same

factor, so that as the temperature goes towards 0 the algorithm searches in a more focused way near the high scoring modules.

T_{init} is an important parameter determining the initial rate of acceptance of detrimental changes of the proposed edge set. It is assigned according to a measurement of the typical scores of modules in random samples of edge sets in the network of interest. t_{init} random edges are selected 100 times and the highest scoring module induced by each of these 100 edge sets is calculated. T_{init} takes the value of $0.2\times$ the difference between the maximum and the minimum of these values. Several different approaches to determining T_{init} were tried, but this method gave the best results in terms of consistency of the final result and number of steps required to reach a stable result.

The algorithm terminates when no scores have changed in the last 10000 steps, or when a certain number of steps have been taken, depending on which occurs first. In many cases, negative scores of reactions are also of interest (in the case study, down-regulation is as informative as up-regulation) so the algorithm can optionally be run with all reaction scores replaced by their negatives (but with metabolite scores remaining negative).

Module statistical significance

The results of module significance tests (see main text) are written to the `qvals` object in the Python .dat file.

References

1. Hucka M, Finney A, Sauro HM, Bolouri H, Doyle JC, Kitano H, Arkin AP, Bornstein BJ, Bray D, Cornish-Bowden A, Cuellar AA, Dronov S, Gilles ED, Ginkel M, Gor V, Goryanin II, Hedley WJ, Hodgman TC, Hofmeyr JH, Hunter PJ, Juty NS, Kasberger JL, Kremling A, Kummer U, Le Novere N, Loew LM, Lucio D, Mendes P, Minch E, Mjolsness ED, Nakayama Y, Nelson MR, Nielsen PF, Sakurada T, Schaff JC, Shapiro BE, Shimizu TS, Spence HD, Stelling J, Takahashi K, Tomita M, Wagner J, Wang J, SBML Forum: **The systems biology markup language (SBML): a medium for representation and exchange of biochemical network models.** *Bioinformatics* 2003, **19**(4):524–531.

2. Finney A, Hucka M: **Systems biology markup language: Level 2 and beyond.** *Biochemical Society transactions* 2003, **31**(Pt 6):1472–1473.

3. Bornstein BJ, Keating SM, Jouraku A, Hucka M: **LibSBML: an API library for SBML.** *Bioinformatics* 2008, **24**(6):880–881.

4. Hagberg AA, Schult DA, Swart PJ: **Exploring Network Structure, Dynamics, and Function using NetworkX** 2008, [[http://math.lanl.gov/ hagberg/Papers/hagberg-2008-exploring.pdf]].

5. Ideker T, Ozier O, Schwikowski B, Siegel AF: **Discovering regulatory and signalling circuits in molecular interaction networks.** *Bioinformatics* 2002, **18 Suppl 1**:S233–40.

6. Kirkpatrick S, Gelatt Jr C, Vecchi M: **Optimization by simulated annealing**. *Science* 1983, **220**(4598):671–680.
