# Supplementary material for: AMBIENT: Active Modules for Bipartite Networks - using high-throughput transcriptomic data to dissect metabolic response
Source: BMC Syst Biol. 2013 Mar 25;7:26. doi: 10.1186/1752-0509-7-26 (PMC3656802; doi:10.1186/1752-0509-7-26)
Supplement: Additional file 2 — Supplementary Figures. Figures S1-S4: diagrams of the positive and negative modules found by both ambient and GiGA. [file 1752-0509-7-26-S2.pdf]

AMBIENT: Active Modules for Bipartite Networks. Using high-throughput transcriptomic data to dissect metabolic response.

Supplementary Figures S1-S4

Each diagram represents a single module inferred by either `ambient` or GiGA, being areas of significantly affected areas of up- and down-regulation in the metabolic network of *S. cerevisiae*. In each diagram, reactions found by both methods are colour-coded, and up- and down-regulated reactions identified by DeRisi and coworkers have black borders. Arrow directions on edges indicate the conventional direction assigned to each reaction; where an arrow points from a reaction to a metabolite, that metabolite is a product of that reaction, and where the arrow points from a metabolite to a reaction it is a substrate. A key can be found below the figure legends.

Figure S1 - statistically significant up-regulated modules found by `ambient`

Shows modules 2, 3 and 4, panels A, B and C respectively, inferred by `ambient` to be up-regulated (module 1 is shown in the main text, Figure 1).

Figure S2 - statistically significant down-regulated modules found by `ambient`

Shows the single down-module inferred by `ambient`.

Figure S3 - statistically significant up-regulated modules found by GiGA

Shows the two modules inferred by GiGA to be up-regulated.

Figure S4 - statistically significant down-regulated modules found by GiGA

Shows the six modules inferred by GiGA to be down-regulated.

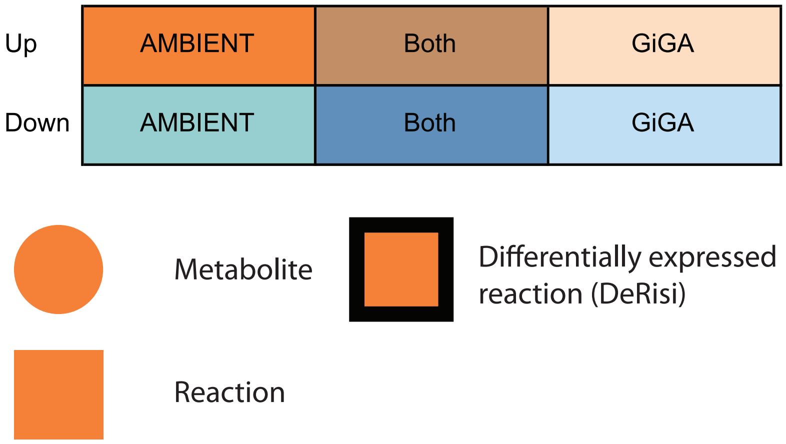

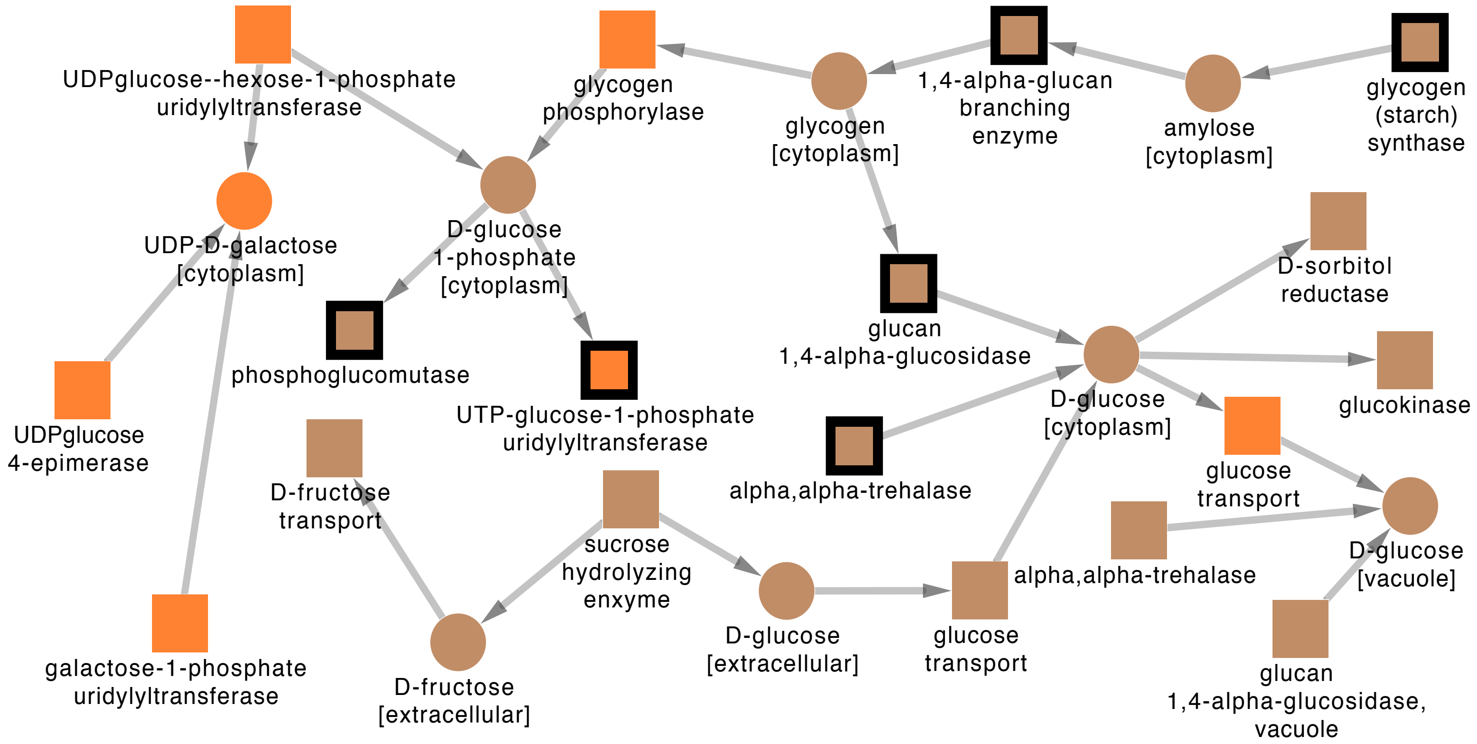

Figure S1A - u2

Figure S1B - u3

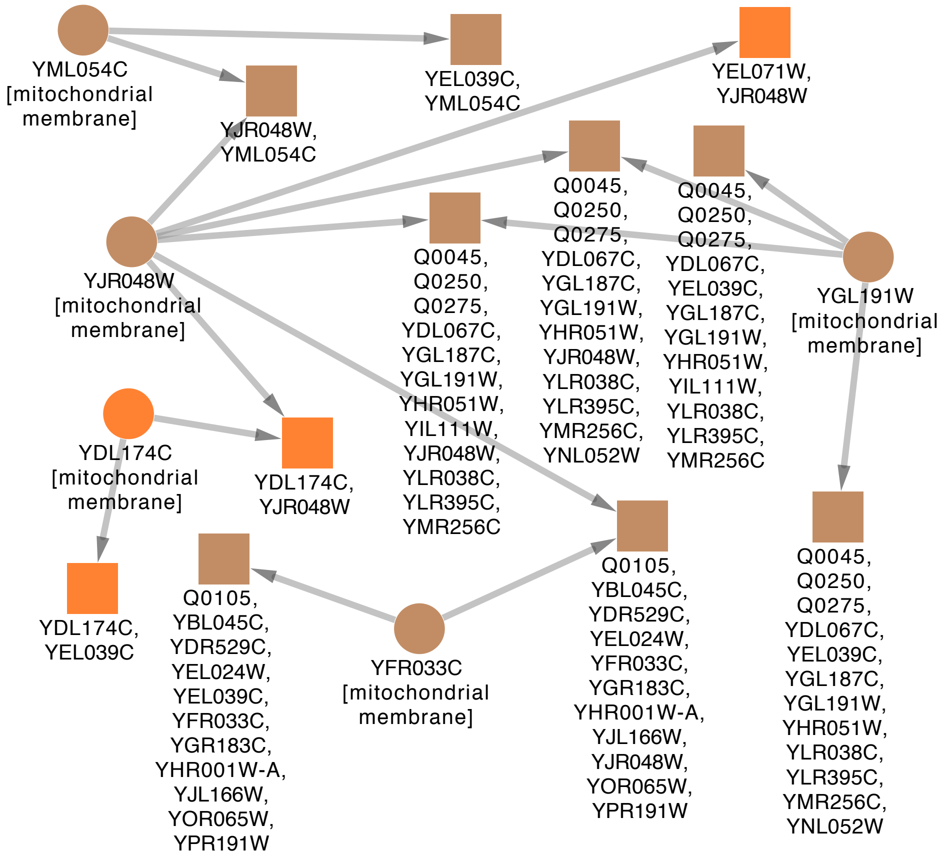

Figure S1C - u4

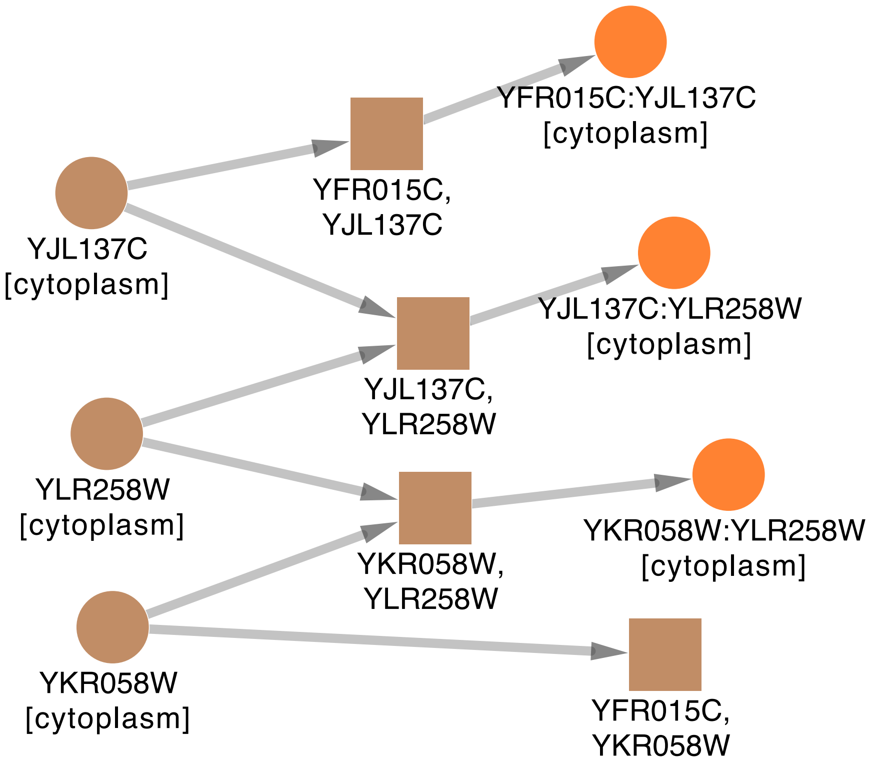

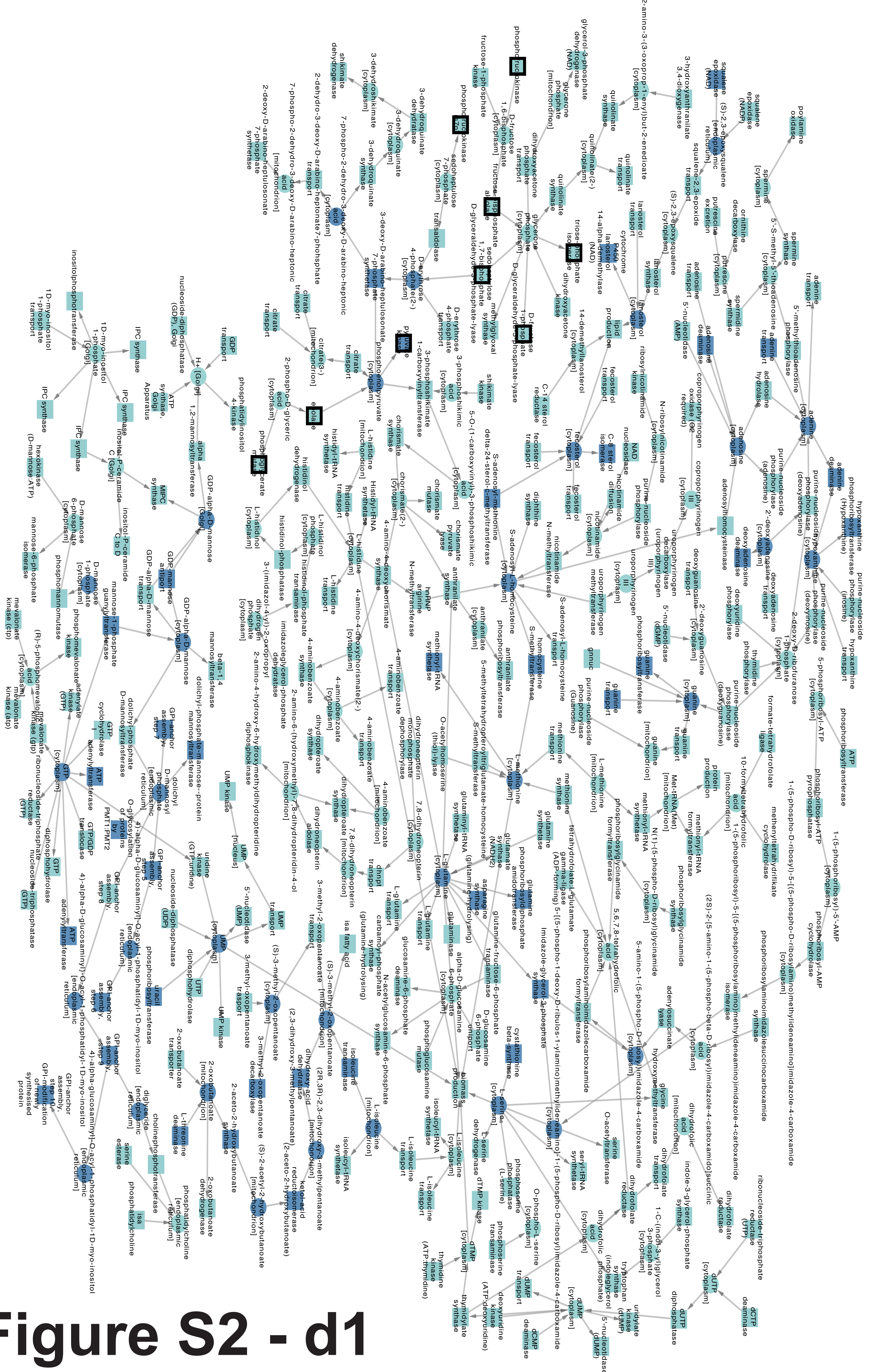

Figure S2 - d1

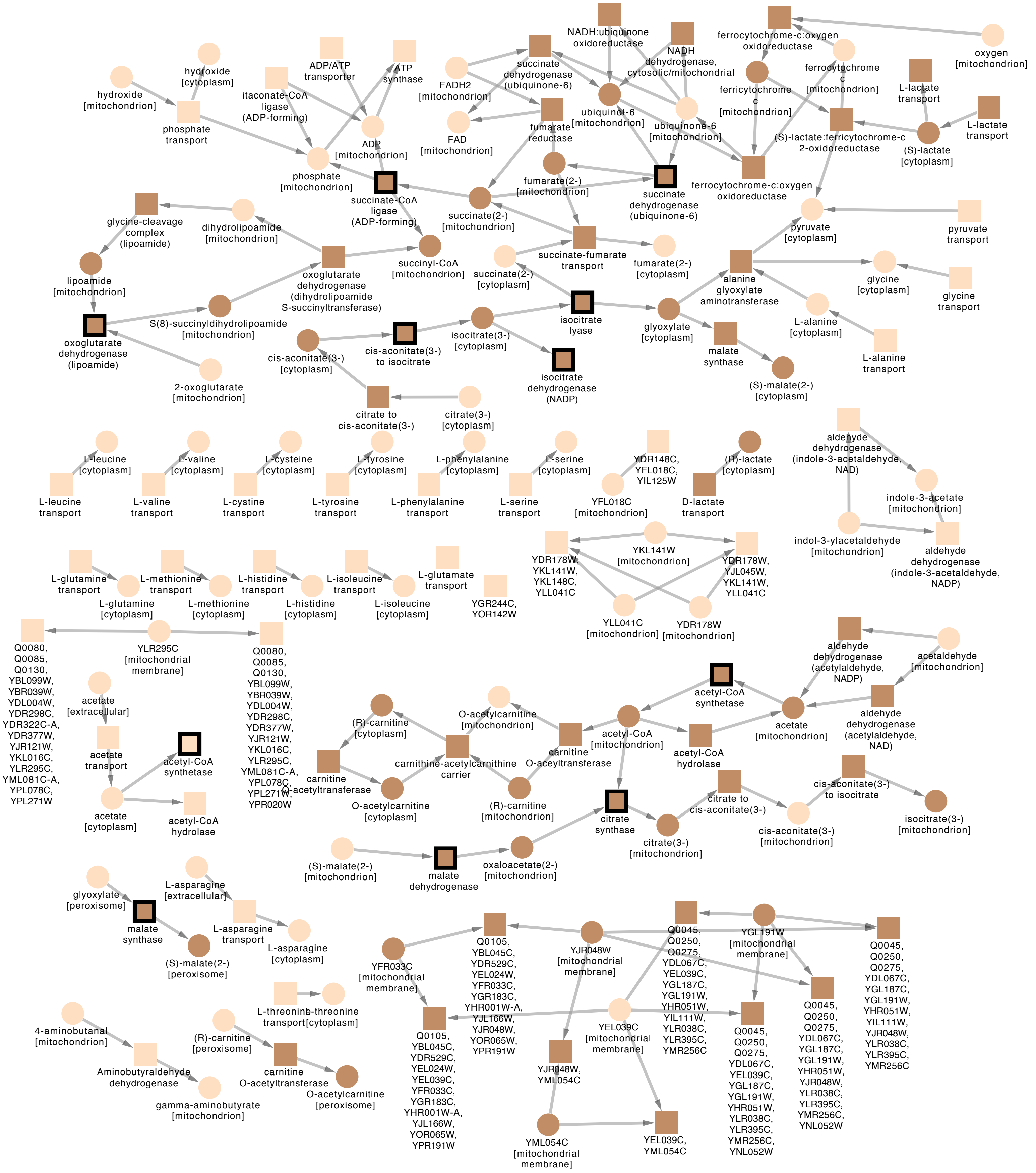

Figure S3A - GiGA up 1

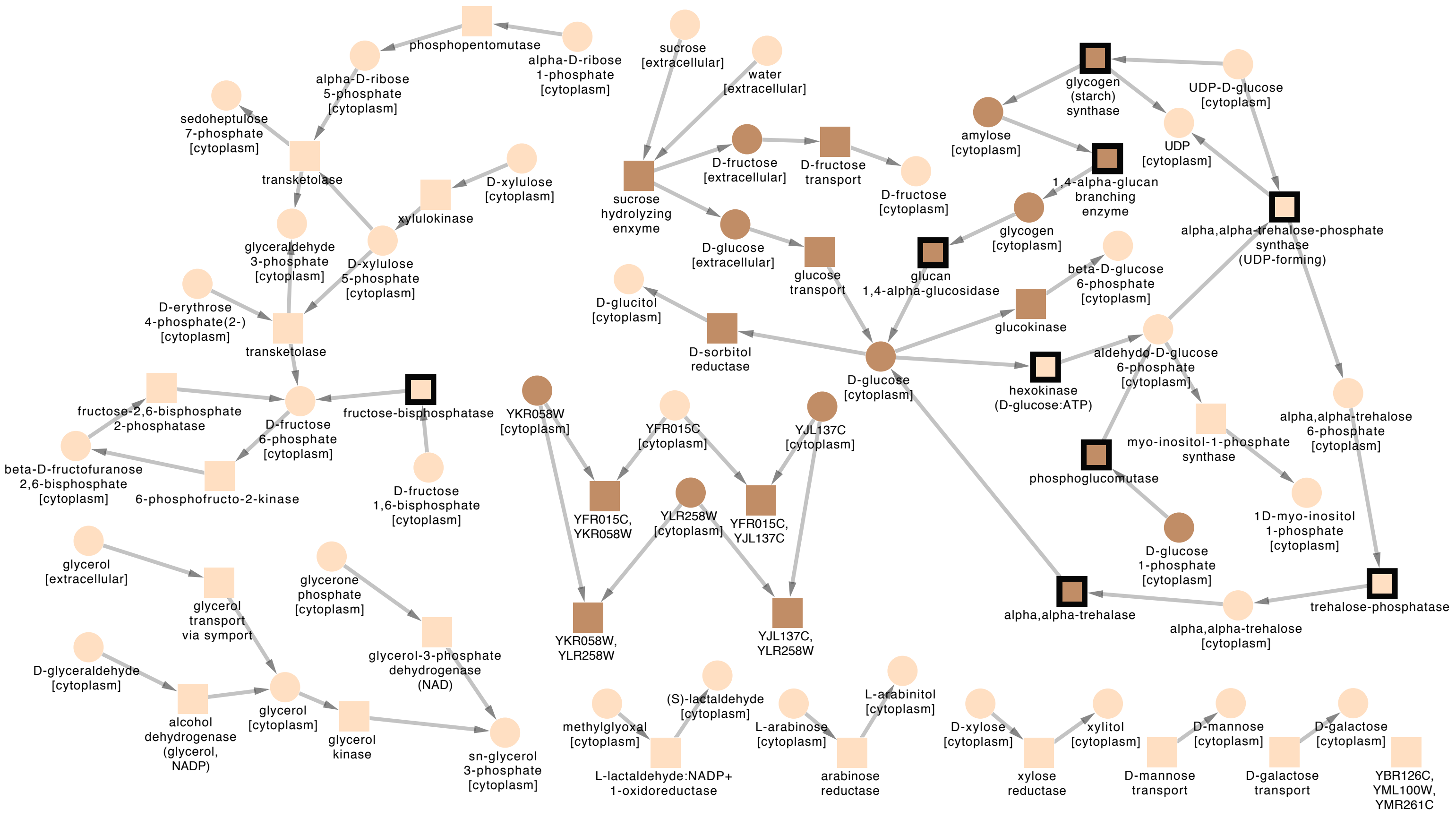

Figure S3B - GiGA up 2

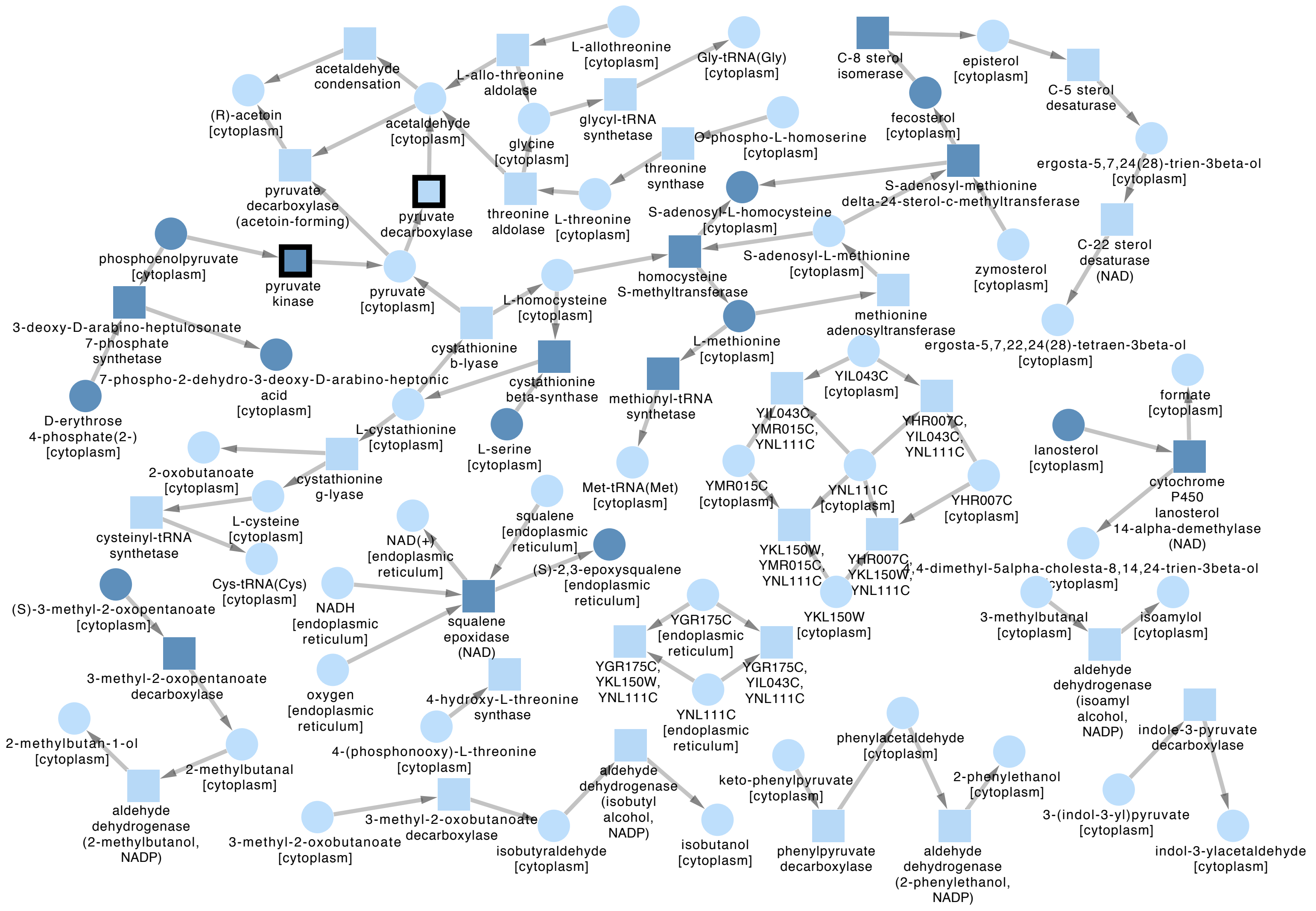

Figure S4A - GiGA down 1

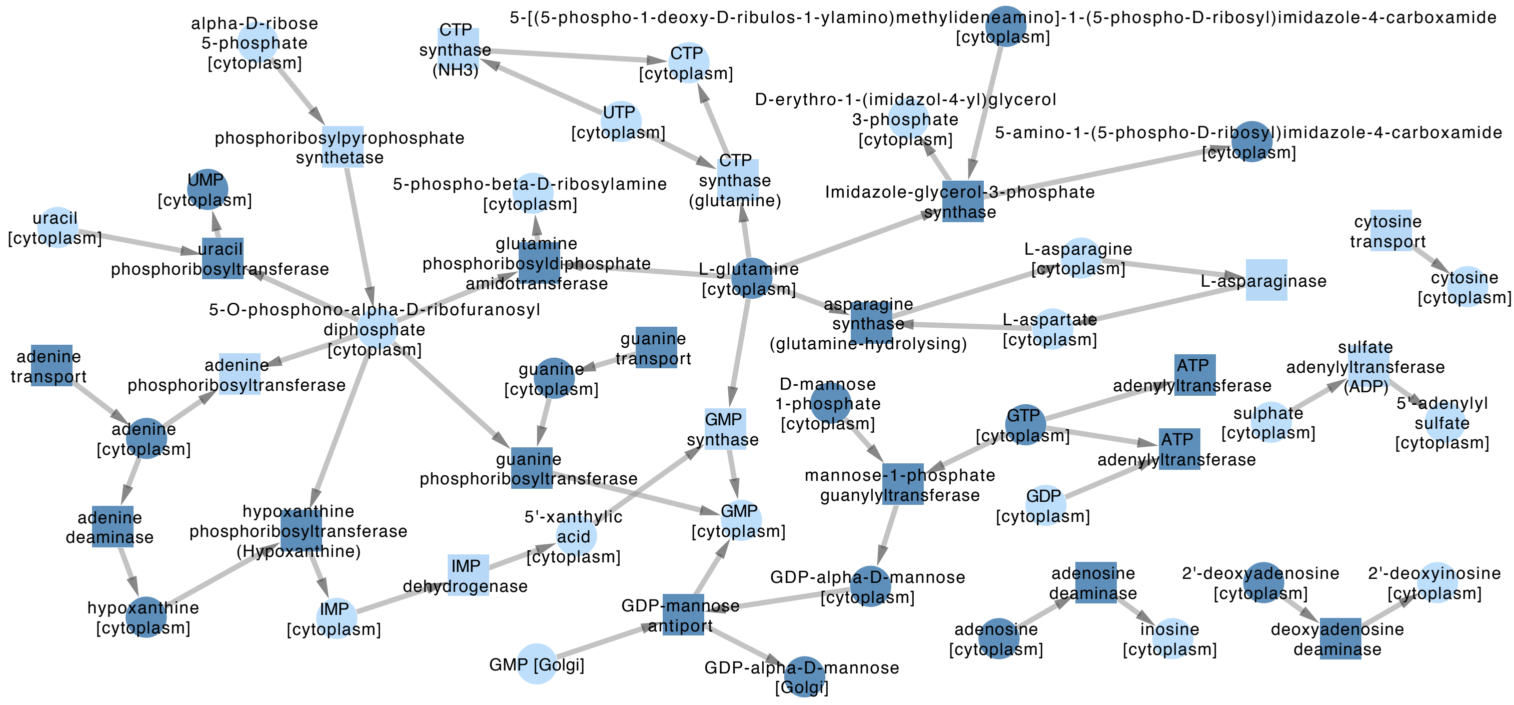

Figure S4B - GiGA down 2

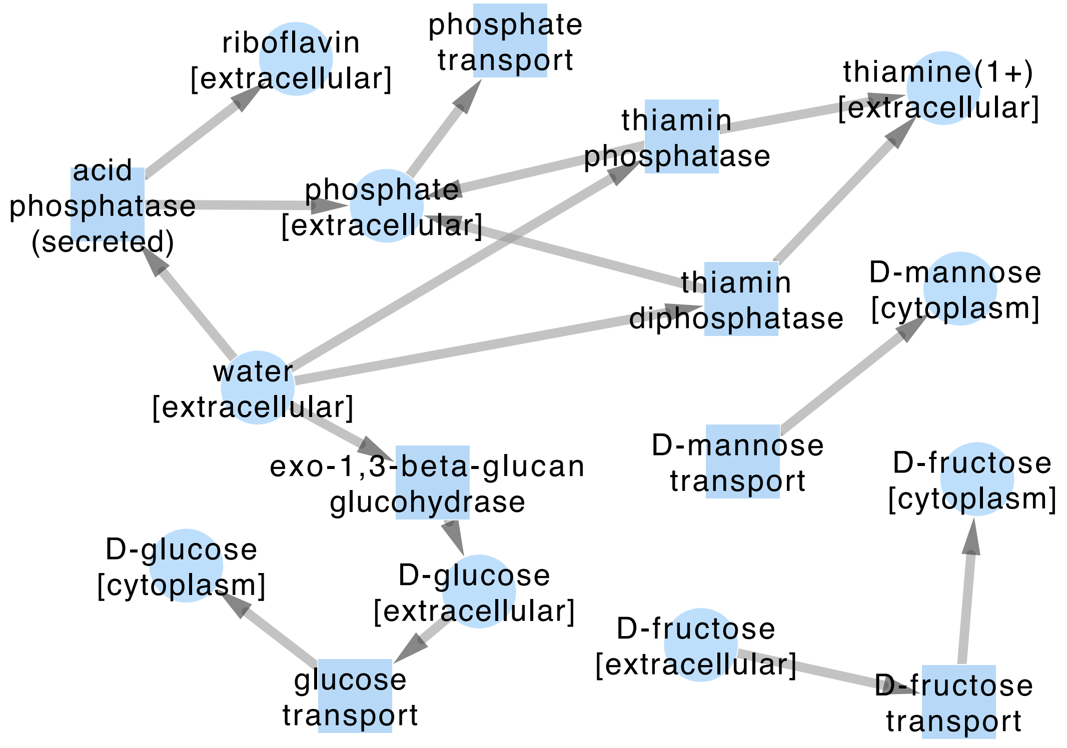

Figure S4C - GiGA down 3

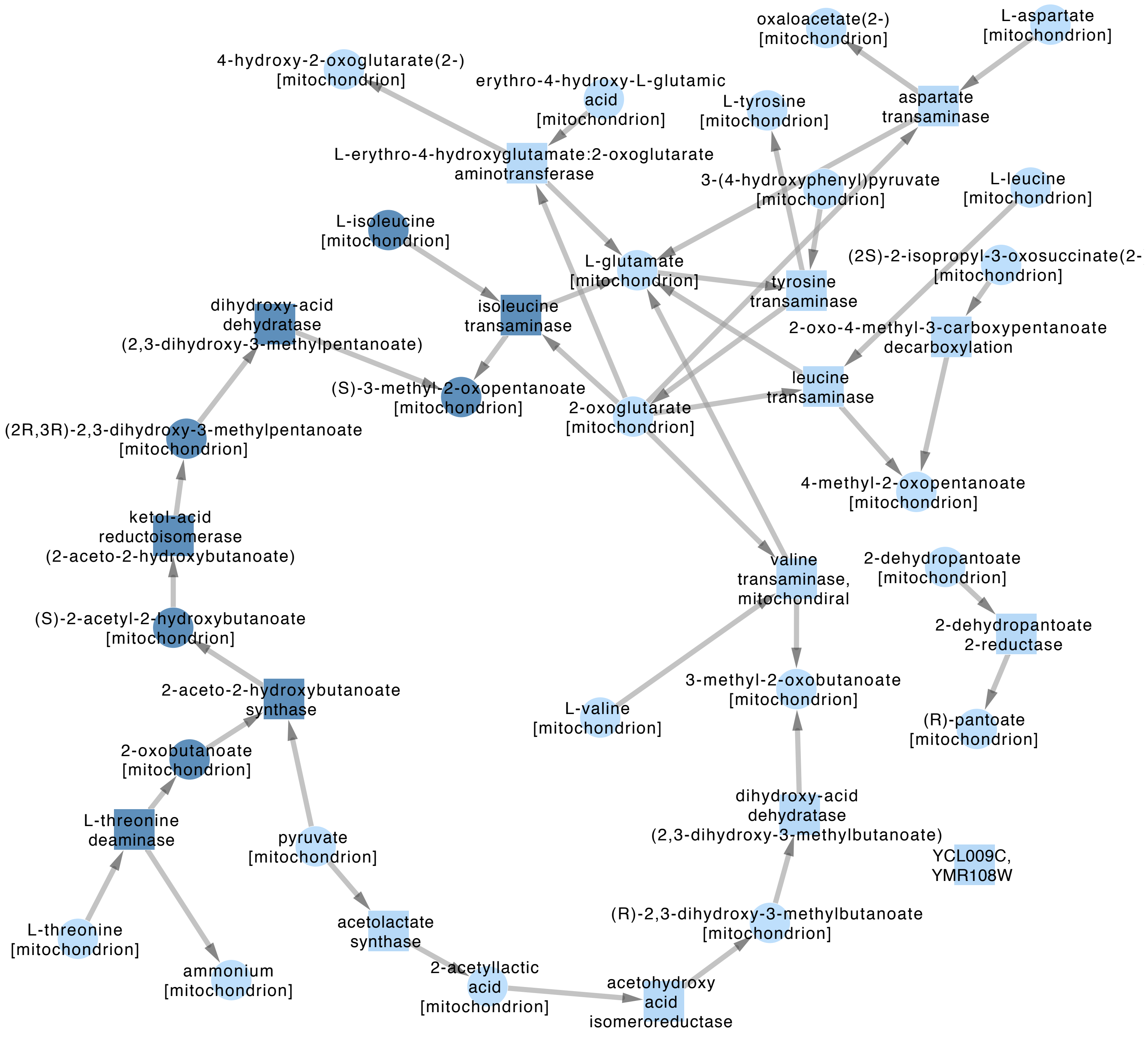

Figure S4D - GiGA down 4

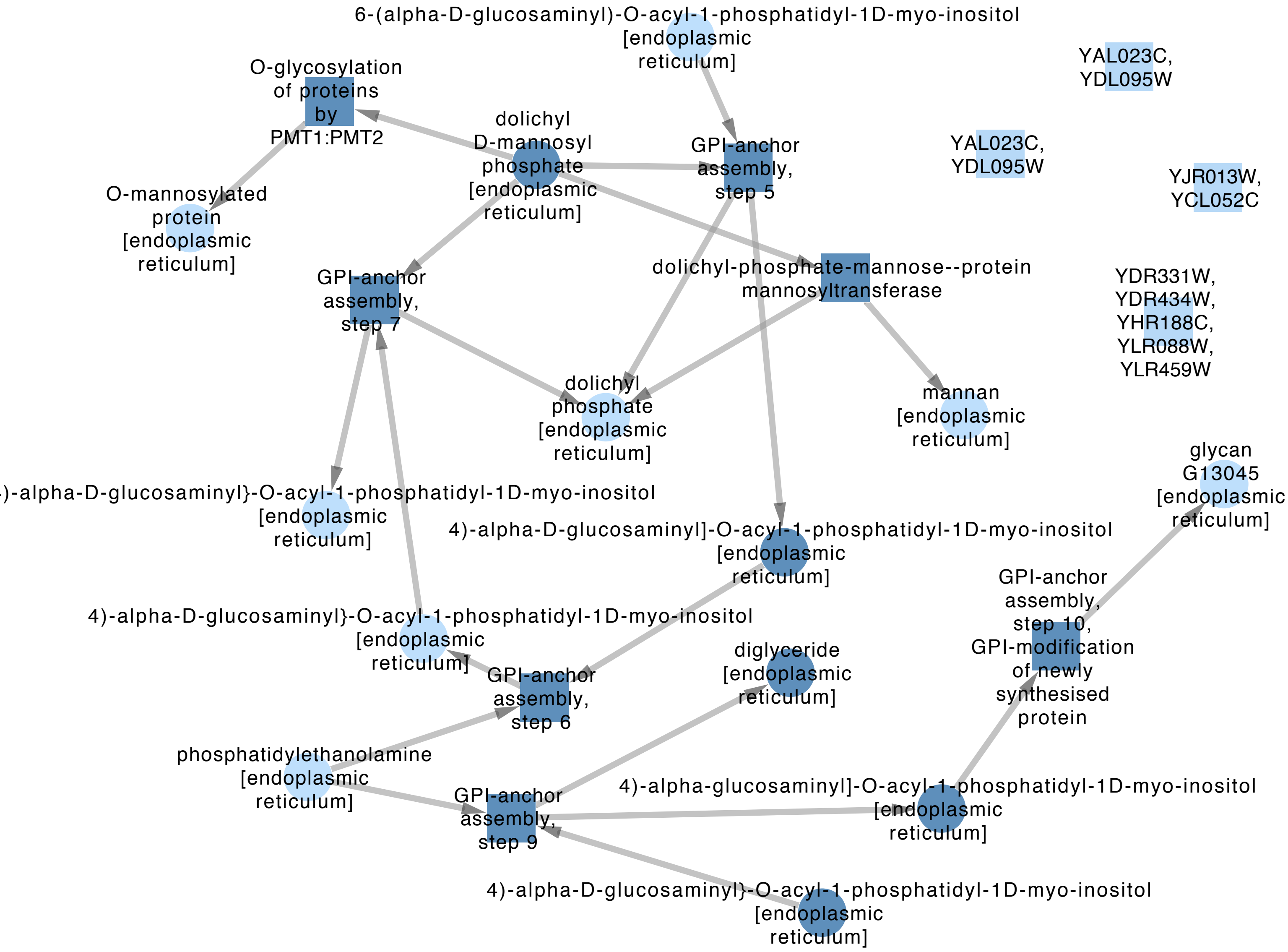

Figure S4E - GiGA down 5

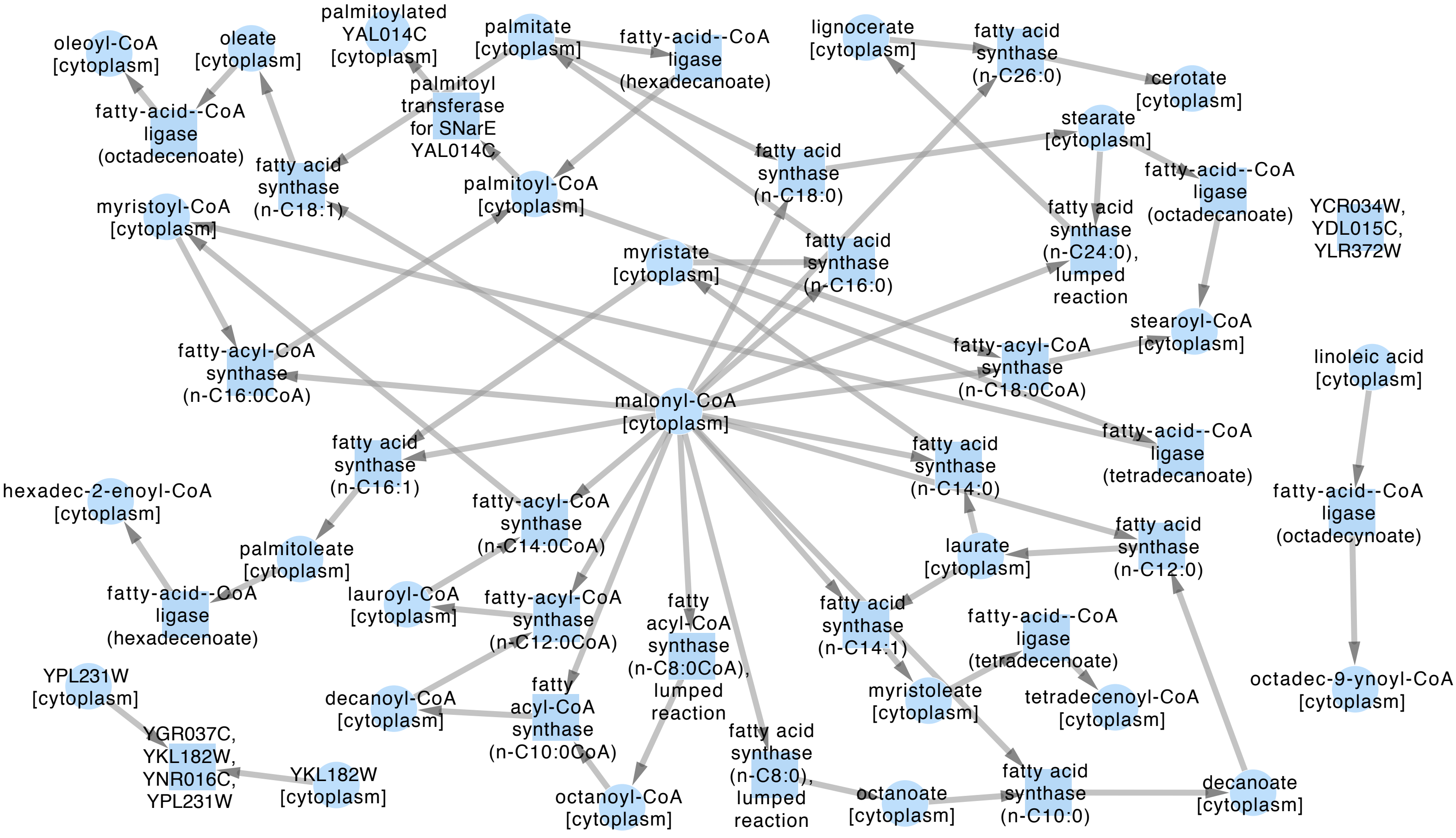

Figure S4F - GiGA down 6
